# Ion-Exchange Chromatography Coupled to Mass Spectrometry in Life Science, Environmental, and Medical Research

**DOI:** 10.1021/acs.analchem.2c04298

**Published:** 2023-01-10

**Authors:** Judith B. Ngere, Kourosh H. Ebrahimi, Rachel Williams, Elisabete Pires, John Walsby-Tickle, James S. O. McCullagh

**Affiliations:** † Department of Chemistry, University of Oxford, Mansfield Road, Oxford OX1 3TA, U.K.; ‡ Institute of Pharmaceutical Science, King’s College London, London SE1 9NH, U.K.

Ion-exchange chromatography (IEC) is a chromatographic technique commonly used for the separation of ions and ionizable molecules. Its direct coupling with mass spectrometry has historically been technically challenging, but the development of online ion-suppression technology has enabled the introduction of commercial ion-chromatography–mass spectrometry (IC-MS) systems. IC-MS can be used to separate, identify, and quantify a very wide range of ionizable compounds in complex samples, including those from inorganic, organic, environmental, and biological origins, and is currently being applied in areas including environmental studies, forensics, medicinal chemistry, cell biology, and metabolomics. In well over 100 publications to date IC-MS has clearly demonstrated unique analytical capabilities compared to complementary and alternative “hyphenated techniques” such as gas chromatography–mass spectrometry (GC-MS) and hydrophilic interaction chromatography coupled to mass spectrometry (HILIC-MS). IC-MS is the last of the main chromatographic types to be coupled directly with mass spectrometry, enabling exciting applications and new research capabilities, especially for life, environmental, and medical sciences. In this review, we explore the development of IC-MS and its separation and analytical characteristics; report on current research applications, compare performance with alternative analytical techniques; and discuss future application areas.

Traditionally, gas chromatography coupled with mass spectrometry (GC-MS), and various types of liquid chromatography coupled with mass spectrometry (LC-MS), such as reversed phase chromatography–mass spectrometry using ion-pairing agents (IP-MS) and hydrophilic interaction chromatography–mass spectrometry (HILIC-MS), have been used for the analysis of samples containing highly polar and ionic compounds. In areas such as forensic science, clinical chemistry, and cell biology, high-sensitivity, chemical selectivity, and high specificity are limiting factors when it comes to the analysis of complex sample matrices. The established analytical techniques referred to often have limitations when ionic and ionizable analytes such as nucleotides, sugar phosphates, phosphorus-containing herbicides, and organic acids are of interest.^1−5^ GC-MS can be used for the analysis of volatile and nonvolatile (derivatized) molecules from a wide range of environmental and biological contexts including water, food, plant extracts, cell extracts, tissues, and biological fluids, but often complex sample preparation is essential with significant modification of sample matrices in favor of selected target molecules, e.g. for the analysis of pesticides, herbicides, toxins, and their metabolic products.^5,6^ Discovery (less targeted) experiments are also possible, e.g., analysis of cellular metabolite profiles,^7,8^ but the extended sample preparation requirements of GC-MS, particularly for the analysis of highly polar and ionic compounds (that often require derivatization), reduces analytical flexibility and applicability.^9^ Liquid chromatography techniques coupled to mass spectrometry, such as IP-MS and HILIC-MS, are commonly used for the analysis of nonvolatile organic ions and highly polar molecules but also have limitations in terms of separation of ionic and ionizable analytes.^9^ For example, anions such as perchlorate, glyphosate, and many metabolites including nucleotides and sugar phosphates^10,11^ are often poorly separated with very high retention factors using HILIC-MS methods.^12,13^ The analytical challenges faced by contemporary GC-MS and LC-MS techniques, in particular for the analysis of ionic and highly polar compounds, highlights a continued need to further improve analytical methods and develop new techniques, particularly for high-sensitivity detection of biomarkers in complex samples, as well as untargeted, discovery-driven applications in environmental and biological studies.

Since the term “ion chromatography” (IC) was first used, it has diversified to represent a range of techniques for the sensitive analysis of ionic and polar compounds. IC represents chromatographic techniques that enable separation of ionic compounds, central to which is ion-exchange chromatography (IEC) but also includes ion-pair chromatography (IP-MS), ion-exclusion chromatography, and a number of ancillary ion-based separation methods.^14^ In this review we focus mainly on high-performance ion-exchange chromatography coupled directly to mass spectrometry as this has been the main separation approach used with hyphenated IC-MS platforms, and the term IC-MS is often used as a synonym for “IEC-MS”. More rarely the term “high performance ion-chromatography–mass spectrometry” (HPIC-MS) is also used. In this review we will use the term IC-MS to refer to the analytical platform and the “ion-exchange chromatography–mass spectrometry” separation mode, unless otherwise stated, in line with common usage in the literature.

IEC should be a highly compatible separation technique for coupling directly to mass spectrometry because it produces separated analytes, already in an ionized form and, therefore, suitable for analysis by mass spectrometry. However, coupling IEC with MS has been experimentally challenging due to the general incompatibility of the mobile phases typically used. High ion-strength and extreme pH eluent conditions interfere with both electrospray ionization (ESI) and atmospheric pressure chemical ionization (APCI) processes that interface with mass spectrometry and can lead to MS detector saturation as well as physical damage to MS instruments over time.^15^ Finding ways to enable MS detection of analytes separated by IEC has been a major technical challenge but has now been largely solved by the use of ion-suppression technology built into the postcolumn eluent flows, which enable online separation and detection by IC-MS.^16,17^ Early applications of IC-MS were largely focused on the analysis of inorganic ions in the context of forensic and environmental studies, but more recently IC-MS has been applied in biological studies, e.g., in the analysis of cells, tissues, and biofluid extracts.

In this review we focus on the emergence of IC-MS which has been enabled by the development of ion-suppression technology. We report on both traditional applications of the method in the past 15 years and highlight important progress in the past 5 years where the technique has been applied in medicinal and biological research. We report evidence that IC-MS provides novel analytical capabilities with potential for exciting development and applications in the future and hope this review stimulates further interest in IC-MS and makes clear the benefits it can provide for a wide range of analytical research.

## Ion-Exchange Chromatography

**Figure 1:**
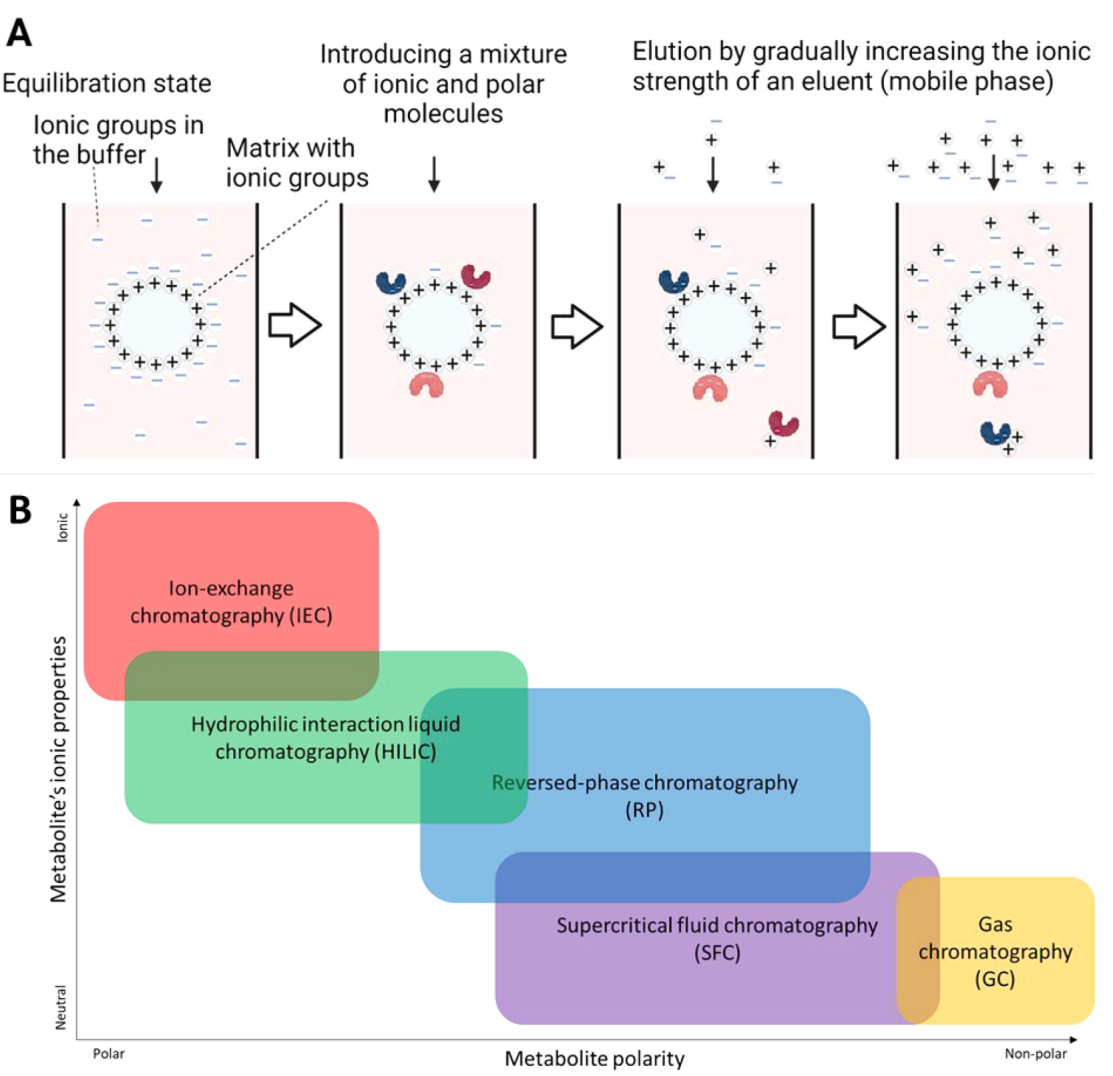
IEC separates highly polar and ionic compounds providing a unique separation space compared to other chromatography types. (A) A schematic representation of the mechanism of anion-exchange chromatography. The column’s stationary phase is positively charged and equilibrated with a mobile phase containing negative ions (e.g., OH^–^ or another anion) at a minimal concentration. When a sample is introduced with negatively charged or polarizable analytes, the charged analytes displace the negative ions (e.g., OH^–^ ions) from the stationary phase and bind instead, therefore being retained. Analyte ions have differential affinity for the stationary phase depending upon their charge; the affinity is directly determined by Coulombic force. The ionic strength of the mobile phase is usually increased for a gradient elution where the concentration of ions in the mobile phase (e.g., OH^–^) is gradually increased until the analyte ions are displaced by the increasing ion concentration in the mobile phase (isocratic elution is also sometimes used). (B) Indicative illustration of chromatographic separation space showing how ion-exchange chromatography extends the separation space beyond reversed-phase chromatography (RP-LC) and hydrophilic interaction liquid chromatography (HILIC) for highly polar and ionic molecules.

IEC is based on the formation of ionic or electrostatic interactions between analyte ions or highly polar molecules and an oppositely charged stationary phase. There are two basic types known as cation-exchange and anion-exchange. In cation exchange mode the stationary phase is negatively charged and analytes that are positively charged interact with the stationary phase. In anion exchange mode the stationary phase is positively charged and interacts with negatively charged analytes. Overall electrostatic charge, charge density, and surface charge distribution of the analyte all play an important role in the mechanism of retention.^18^ Typically, during chromatographic separation, the ionic strength of the mobile phase eluent is gradually increased to displace charged analytes that are ionically interacting with the stationary phase. The elution times of individual ions are determined by the strength of the ionic interactions between the analyte and stationary phase (Figure 1A). Hence, coupling IC with mass spectrometry, especially high-resolution instruments, enables ions to be both separated and have their mass-to-charge ratio measured on a continuous basis. There is some overlap with HILIC-MS and IP-MS in terms of compatible analytes, but the mechanism of retention in IEC leads to a unique analyte separation profile, particularly for ionic compounds as illustrated in Figure 1B.

## Hyphenation of IEC with MS

**Figure 2:**
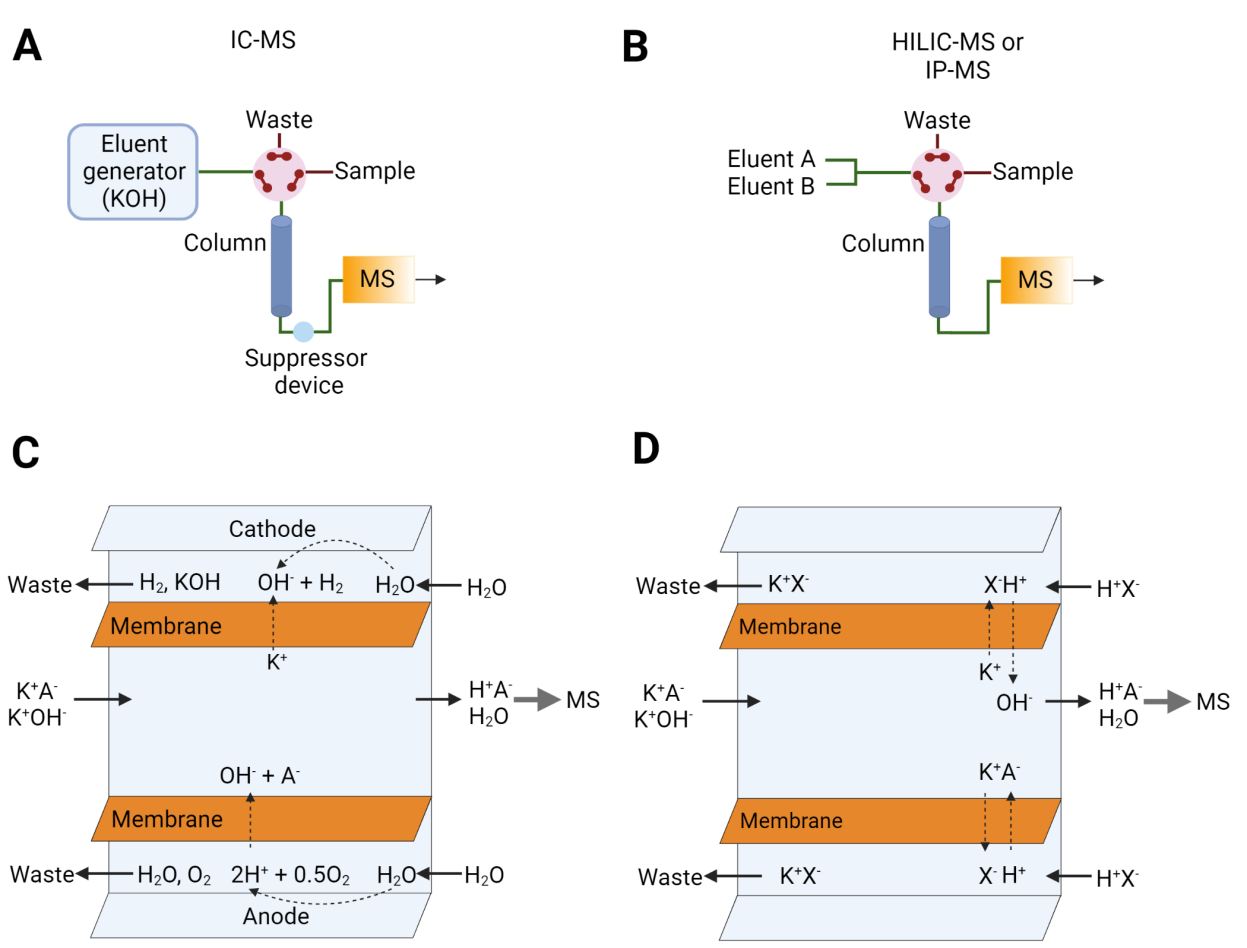
Hyphenation of IC to MS using ion suppression devices. (A) A schematic representation of IC-MS compared to (B) HILIC-MS or IP-MS. (C) A schematic representation of the mechanism of electrolytic ion-suppression and (D) and chemical ion-suppression. The orange film represents a semipermeable membrane through which ions are transported to or from the sample.

Prior to the development of online hyphenated systems coupling IC with MS, analysis of IC separated compound by MS was a labor-intensive analytical process incorporating the collection of individual eluent fractions offline and their individual analysis by MS. Three approaches have been used to enable direct coupling with mass spectrometry:^19^ (i) direct coupling using MS-compatible volatile solvents, (ii) two-dimensional liquid chromatography (2D-LC), and (iii) use of ion-suppression technology. Direct coupling using MS-compatible volatile solvents is suitable only for a smaller fraction of analytes as it is limited to analytes that elute by ion-exchange mechanisms using volatile mobile phases.^13,20−24^ Two-dimensional liquid chromatography (2D-LC) provides access to a wider range of analytes. It involves the injection of fractions from the eluent of a first ion-exchange separation into a second separation dimension (often based on reversed-phase chromatography) where separation of analytes from the nonvolatile mobile phase components takes place as well as potentially further separation of analytes in the fraction.^25−27^ Its high selectivity coupled with online interfacing of conventional ion-exchange chromatography with mass spectrometry provides a powerful analytical platform. However, the system is more complex and expensive than a typical stand-alone IEC system and typically has reduced sensitivity due to sample losses in the second separation dimension. Limitations linked to these first two methods (i and ii) have largely been addressed by the development of ion-suppression approaches that enable direct coupling of conventional IEC systems with mass spectrometry without reducing the mobile phase choice or requiring additional separation dimensions (Figure 2A,B). Some of the earlier reports of ion-suppression technology go back to 1975, but in 1990 work by Conboy et al. led to significant developments.^28^

Currently there are two types of ion-suppressors used in online IC-MS: electrolytic suppressors and chemical suppressors. Both are capable of continuous online substitution of mobile phase ions that are not compatible with MS. Most provide H^+^ or OH^–^ ions (depending on the ion mode of IEC separation) which, combined with the removal of the salt counterion (typically K^+^ or Cl^–^), form an aqueous mobile phase eluent at neutral pH. Figure 2C,D illustrates the working principles behind electrolytic and chemically regenerated suppressor devices. In a series of studies, Karu et al. investigated the use of each suppressor for coupling IEC to mass spectrometers and the effect of the composition of mobile phase on stability and performance of the analytical method.^16,17,29,30^ They compared the application of electrolytic and chemical suppressors for the analysis of selected organic acids of relevance in pharmaceutical industry applications including flufenamic acid, mefenamic acid, and fenbufen. They showed that application of electrolytic suppressors with aqueous eluents generally led to more robust ESI-MS detection.^29^ For example, while both types of ion suppression techniques had comparable limits of detection (<50 ng/mL), the peak area percentage relative standard deviation (%RSD) values were generally 1.5–3 fold lower for electrolytic suppression methods compared to chemical suppression.^29^ On the other hand, it was found that chemical suppression for analysis of aqueous/organic eluents led to a more uniform and lower baseline and less gradient drift compared to electrolytic suppression.^16,30^

## IC-MS Applications

IEC provides sensitive and robust separation and quantification of ionic and highly polar inorganic and organic compounds from synthetic, environmental, and biological origins. High-resolution mass spectrometry (HRMS) provides sensitive and selective detection for chemical characterization and structural elucidation. Coupling both instruments directly (IC-MS) enhances the analytical capabilities of both techniques. Table 1 provides a selection of studies which demonstrate the comparative performance of IC-MS with other techniques, including GC-MS, RP-MS, and HILIC-MS covering the last 20 years but focusing mainly on recent studies. These studies illustrate both the breadth of applications and competitive limits of detection and quantification provided by IC-MS. Additionally, they demonstrate that electrolytic ion-suppression is currently the preferred choice of ion-suppression method for IC-MS. The selectivity of IEC for polar and ionizable analytes (anions or cations) is particularly beneficial for the analysis of complex samples as it reduces the potential for matrix interference which facilitates increased analytical stability compared to alternative LC-MS techniques.^31^ Although IEC was traditionally a technique applied in inorganic chemistry contexts, its coupling with mass spectrometry detection has broadened its applications to include organic and biological analytes in a wider range of application areas.

**Table 1:** Comparison of the Historical Analytical Performance of IC-MS with Other Chromatographic Methods from 1997 to 2020^a^

Sample Type	Compound	Approach	Suppressor Type	Method	LOD	LLOQ	%RSD/ % CV	Ref	Year
Urine	APAs and AMPAs	Targeted	No suppressor	IC-MS	0.3–2 ng/mL	1–60 ng/mL	11–14	(13)	2019
Urine	MPA	Targeted	No suppressor	IC-MS	4 ng/mL	10 ng/mL	5–12	(33)	2017
Urine	MPA	Targeted	N/A	GC-MS	57 ng/mL	nr	nr	(41)	1997
PlasmaandUrine	MPA	Targeted	N/A	HPLC-UV	10000 ng/mL	100–12000 ng/mL	nr	(42)	2001
Latent finger-marks and swabbed hand sweat	Gunshot residue (e.g., nitrate, benzoate, perchlorate)	Targeted/untargeted	Chemically	IC-MS	0.3–50 ng/mL	1–30 ng/mL	0.4–10	(43)	2019
Latent finger-marks and swabbed hand sweat	Gunshot residue (DPA)	Targeted	N/A	RP-MS	0.29–0.46 ng/mL	2.9–11.6 ng/mL	nr	(44)	2016
Latent finger-marks and swabbed hand sweat	Gunshot residue	Targeted	N/A	GC-MS	1–50 ng/mL	nr	nr	(45)	2019
Finger-marks	Gunshot residue	Targeted	N/A	RP-MS	nr	0.01–26 ng	4–17	(46)	2015
Finger-marks	Smokeless gunpowder	Targeted	N/A	(CEC)-UVor MS	(DL) 1000–3000 ng/mL	nr	nr	(47)	2012
Plant-derived commodities	Fosetyl and phosphonic acid	Targeted	No suppressor	IC-MS	nr	10 ng/g	1.2–17.8	(59)	2018
Water and soil	Glyphosate and phosphonic acid	Targeted	N/A	GC-MS	3 ng/g	6 ng/g	8–23	(60)	2000
Food commodities	Fosetyl and phosphonic acid	Targeted	N/A	LC-MS	nr	10–50 ng/g	nr	(61)	2019
Food of animal origin	Glyphosate	Targeted	Electrolytically	IC-MS	nr	4.3–9.26 ng/g	<20	(53)	2019
Wheat grain	Glyphosate	Targeted	N/A	RP/GC-MS/HPLC-FD	nr	5 ng/g		(62)	2007
Soy-based infant formula	Glyphosate	Targeted	N/A	HPLC-FD	nr	20 ng/g	nr	(63)	2018
Baby food commodities	Glyphosate, MPA, chlorate, perchlorate, etc	Targeted	Electrolytically	IC-MS	nr	2–5 ng/mL	<20	(56)	2020
Milk-based baby foods	glyphosate and glufosinate	Targeted	N/A	RP-MS	1–2 ng/g	nr	nr	(126)	2018
Drinking waterand soil leachate	Carboxylic acids	Untargeted	Electrolytically	IC-MS	18–60 ng/mL	12–176 ng/mL	nr	(75)	2007
Effluent waters	Haloacetic acids	Targeted	Electrolytically	IC-MS	0.1–0.7 ng/mL	nr	0.5–19	(76)	2009
Tap, river, effluent, and influent water	Dialkyl phosphinate acids (DPAs) and hydrolysates of aluminum DPs (ADPs)	Targeted	Electrolytically	IC-MS	0.001–0.003 ng/mL	0.003–0.01 ng/mL	<20	(77)	2015
Activated sludge	Monosaccharides	Targeted	Electrolytically	IC-MS	0.34–2.15 ng/mL	nr	<4	(79)	2020
Blood plasma	Zoledronic acid	Targeted	Electrolytically	IC-MS	0.2 ng/mL	nr		(84)	2020
Plant	Phosphorylated metabolites	Untargeted	Electrolytically	IC-MS	10–250 nM	nr	93–110	(15)	2005
Plant root exudates	Organic acids	Untargeted	Electrolytically	IC-MS	nr	5 ng/mL	nr	(102)	2020
Plant tissues	Organic acids	Untargeted	N/A	HILIC-MS	1–30 μg/mL	3–100 μg/mL	nr	(127)	2009
Plasmaand urine	Perchlorate, thiocyanate, rodenticides, iodine, and nitrate	Targeted	Electrolytically	IC-MS	0.25–1 ng/mL	1–10 ng/mL	8	(34−39)	2005/06/09/09/09/09
Blood	Rodenticides	Untargeted	N/A	RP-MS	0.5–1 ng/mL	nr	15–20	(128)	2015
Plasma	Rodenticides, drugs, natural products	Untargeted	N/A	RP-MS	5–25 ng/mL	2.5–25 ng/mL	nr	(129)	2010

a nr, not reported; CEC, capillary electrochromatography.

### Forensic Science and Toxicology

**Figure 3:**
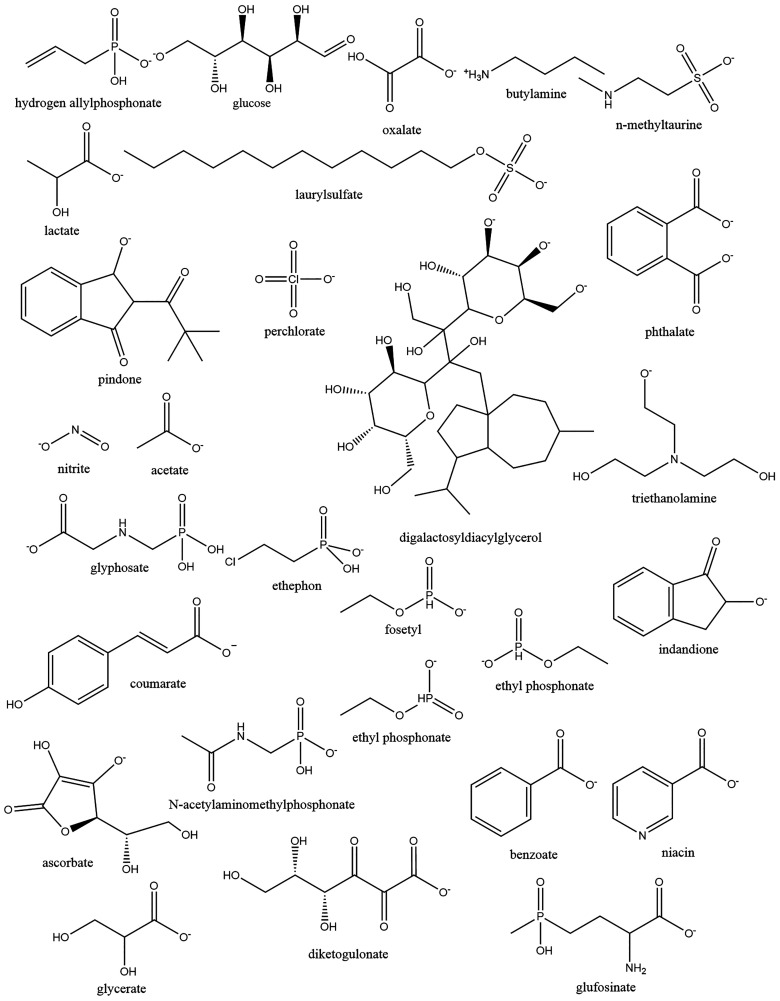
Some examples of ions previously characterized by IC-MS across a range of studies.

There is growing need for more sensitive, selective, and robust molecular analysis techniques in forensics and clinical chemistry. For example, identification of a wider range of biomarkers in body fluids; DNA residue analysis; drugs of abuse identification; gun and explosive residue analysis; and toxic compounds such as pesticides, herbicides, and nerve agents and their metabolites. From a forensic and public-health standpoint, it is advantageous to be able to detect and identify compounds of interest in a wide range of sample types with high sensitivity and selectivity. Hence, there has been a strong interest in the use of chromatographic techniques coupled with HRMS.^32^ IC-MS has two analytical qualities of particular interest in forensic science and toxicology: (i) analytes are often ionic or highly polar in nature, and (ii) IC-MS provides methodological robustness and simplicity for analysis of complex sample types, e.g., biofluids such as blood plasma and urine and environmental samples such as soils. These capabilities were thoroughly reviewed in 2014.^32^Table 2 provides selected examples of applications including analysis of highly polar organophosphorus markers relating to nerve agents,^13,33^ perchlorate and thiocyanate,^34,35^ rodenticides,^36−38^ and biomarkers including iodine and nitrate^35,39^ in body fluid extracts such as plasma and urine, while Figure 3 exemplifies some chemical structures analyzed by IC-MS. Here, we focus on more recent examples of IC-MS for analysis of ionic and polar molecules related to forensic science applications.

**Table 2:** Indicative List of the Compound Types Characterized by IC-MS across a Range of Research Applications, Excluding Intracellular Metabolites

Subgroup	Example	Field of Study	Ref
Phosphinate	Alkylphosphonic acids like methyl, ethyl and propyl	Forensic science, clinical chemistry and diagnostics	(13, 33)
Phosphinate	Methylalkylphosphonic acids	Forensic science	(13)
Phosphinate	Glyphosate	Food chemistry	(50, 52)
Phosphinate	Glufosinate	Food chemistry	(52)
Phosphinate	N-Acetyl glufosinate	Food chemistry	(52)
Phosphinate	N-Acetyl glyphosate	Food chemistry	(50)
Phosphinate	Ethephon	Food chemistry	(51)
Phosphinate	N-Acetyl aminomethylphosphonic acid (AMPA)	Food chemistry	(50)
Phosphinate	2-Hydroxyethylphosphonic acid	Food chemistry	(50)
Phosphinate	Dialkylphosphinate acids	Environmental science and technology	(77)
Halogenated	Pentachlorobenzene	Food chemistry	(130)
Halogenated	Hexachlorobenzene	Food chemistry	(130)
Carboxylic acid	Benzoate	Forensic science	(131)
Carboxylic acid	Formate	Forensic science, environmental science and technology	(132)
Carboxylic acid	Glycerate	Forensic science	(131)
Carboxylic acid	Acetate	Forensic science	(131)
Carboxylic acid	Ascorbate	Forensic science	(133, 134)
Carboxylic acid	Monohydrated diketogulonic acid	Forensic science	(133)
Carboxylic acid	Oxalate	Forensic science, food chemistry, environmental science and technology	(131−133, 135)
Carboxylic acid	Phthalate	Forensic science	(131)
Carboxylic acid	Threonate	Forensic science	(133, 134)
Carboxylic acid	Niacin	Food chemistry	(136)
Carboxylic acid	Maleic	Environmental science and technology	(132)
Carboxylic acid	Tartaric	Environmental science and technology	(132)
Carboxylic acid	Haloacetic acid	Environmental science and technology	(64)
Carboxylic acid	Glufosinate	Food chemistry	(50, 56)
Amine	Monomethylamine	Pharmaceutical industry	(83)
Amine	Ethanolamine	Pharmaceutical industry, environmental science and technology	(83, 137)
Amine	Butylamine	Pharmaceutical industry	(83)
Amine	Triethanolamine	Pharmaceutical industry, environmental science and technology	(83, 137)
Surfactant	Lauryl sulfate	Pharmaceutical industry, environmental science and technology	–
Surfactant	Laureth sulfate	Pharmaceutical industry, environmental science and technology	–
Surfactant	Taurates	Pharmaceutical industry, environmental science and technology	–
Surfactant	Sulfosuccinates	Pharmaceutical industry, environmental science and technology	–
Aromatic (e.g., pesticides, rodenticides)	Chlorophacinone	Environmental science and technology, clinical chemistry and diagnostic	(36)
Aromatic (e.g., pesticides, rodenticides)	Indandione	Environmental science and technology, clinical chemistry and diagnostic	(37)
Aromatic (e.g., pesticides, rodenticides)	Pindone	Environmental science and technology, clinical chemistry and diagnostic	(38)
Nitrogen oxoacid	NO_2_^–^, NO_3_^–^	Forensic science, food chemistry	(131, 134, 135, 138)
Halogen oxoacid	ClO_2_^–^, ClO_3_^–^, ClO_4_^–^	Forensic science, food chemistry, clinical chemistry and diagnostics	(34, 35, 50, 56, 131, 134, 138, 139)
Phosphate oxoacid	PO_4_^–^, PO_3_^–^	Forensic science, Environmental Science and technology	(138, 140)
Sulfur oxoacid	SO_4_^2–^, S_2_O_3_^2–^	Forensic science	(131, 138)
Other	OCN^–^, Cl^–^, I^–^, HS^–^, SCN^–^, Cr (VI), arsenic	Forensic/environmental science and technology, clinical chemistry and diagnostics	(56, 69, 131)

Highly polar alkylphosphonic acids (APAs), less polar alkylmethylphosphonic acids (AMPAs) with hydrophobic fragments, and methylphosphonic acid (MPA) are examples of biomarkers of organophosphorus nerve agents. These molecules are ionic and highly polar and not well-characterized by standard RP-MS or HILIC-MS without derivatization.^13^ Additionally, derivatization of these molecules from complex environmental or biological matrices is challenging, which limits the analytical range and sensitivity of the methods, in part due to the fact that polar compounds do not always dissolve well in the organic solvent used.^13^ To overcome these challenges, Baygildiev et al. used an anion-exchange column for simultaneous identification and characterization of a wide range of underivatized APAs and AMPAs by IC-MS in urine.^13^ They characterized 18 different APAs and AMPAs with lower limit of detection (LLOD) ranging from 0.3 to 20 ng/mL, low limits of quantification (LLOQ) ranging from 1 to 60 ng/mL, accuracy of 1–12%, and intraday and interday precision RSDs of maximum 11% and 14%, respectively. The benefits of IC-MS in this context eliminated the need for derivatization, enabling samples to be analyzed from aqueous solution directly. This made sample preparation much more straightforward, rapid, and robust. In addition, simultaneous characterization of a broad range of APAs and AMPAs was achieved with high sensitivity. In a separate study, Baygildiev et al.^33^ used IC-MS for the rapid analysis and identification of MPAs in rat urine. Using IEC, they separated a wide range of MPAs. Conversely, analysis of the same compounds using RP-LC resulted in overlapping chromatographic peaks and reduced sensitivity.^40^ Baygildiev et al. reported LLOD and LLOQ of 4 ng/mL and 10 ng/mL, respectively.^33^ This significantly improved upon the reported detection limit of approximately 57 ng/mL by GC-MS^41^ and 10,000 ng/mL using HPLC and UV–visible spectroscopy.^42^ Rapid identification of MPAs, i.e., less than 7 min per sample, was achieved without the need for derivatization using IC-MS, hence offering an attractive alternative to time-consuming traditional GC-MS methods.^33^

Inorganic ions such as nitrate, sulfate, or chlorate and organic acids such as acetate and lactate are examples of ionic post-blast residues present in environmental and biological samples. Their detection is important to provide information about the type and extent of a blast event and gunshots. Gallidabino et al.,^43^ using chemical suppression, developed an IC-MS method with two objectives: (i) compatibility with extraction/sampling methodologies used in many forensic science applications, i.e. based on ethanol, isopropanol, or their 50–50 (v/v) mixture with water, and (ii) simplicity.^43^ The method was suitable for untargeted analysis of samples leading to correct classification of gunshot residues from three different ammunition types.^43^ To achieve this, they evaluated a 50:50 (v/v) ethanol/water mixture as the IC eluent, thereby eliminating the need for auxiliary postcolumn infusion to facilitate gas-phase transfer. They analyzed several anions, including nitrate, benzoate, and perchlorate, to test selectivity, LLOD, and LLOQ. The LLODs and LLOQs for most of the anions were in the range of 0.3–50 ng/mL and 1–30 ng/mL, respectively. Additionally, retention time %RSDs were less than 0.4 and 10, respectively. The LLODs, LLOQs, and %RSD values are generally within experimental error and are better or similar to those reported for characterization of gunshot residues using other LC-MS or GC-MS methods (Table 1).^44−47^

### Food Chemistry

One of the analytical challenges in food chemistry and related industries is the ability to detect and identify a range of residual organic and inorganic molecules in food samples to ensure compliance with regulatory limits. In this respect, IC-MS was adopted relatively rapidly in food science research and applications and related industries. Applications which saw the early adoption of IC-MS included detection of pesticide levels in commercial fruits, vegetables, and beverages;^48−54^ sugar concentrations in dried bean crops;^55^ herbicides in baby food commodities;^56^ 1-hydroxyethylidene-1,1-diphosphonic acid in uncooked food;^57^ halogens and sulfur in pet foods;^58^ and fosetyl and phosphonic acid in plant-derived matrices.^59^ These studies demonstrate broad applications in food science largely driven by the competitive analytical performance, and sample preparation simplicity required by, IC-MS, combined with the polar and ionic nature of the analytes (Table 1). The enhanced analytical performance is exemplified in a study by Bauer and co-workers who used IC-MS for the detection of fosetyl and phosphonic acid herbicides in plant-derived commodities.^59^ They reported detection of both compounds with a LLOQ at a level of 10 ng/g with high recovery rates (76–105%) and reproducibility (%RSD of 1.2–17.8%). The reported LLOQ values are lower than or within the reported range for analysis of fosetyl and phosphonic acid in dry matrices using GC-MS or RP-MS^60,61^ (Table 1). Similarly, Chiesa et al.^53^ applied IC-MS for detection and characterization of glyphosate, an herbicide, and its metabolites found in animal-derived food products. Development of a highly sensitive analytical method for identification of glyphosate is important because it has been reported as a carcinogen according to the International Agency for Research on Cancer.^53^ The IC-MS method developed was highly sensitive, with a LLOQ in the range of 4.3–9.26 ng/g, and the precision (coefficient of variation or CV%) was between 2 and 13. These values are lower or the same as the LLOQ values of 10–100 ng/g and CV% of 4–12 reported for other LC-MS and GC-MS methods used for analysis of wheat grain samples^62^ (Table 1). In another study, Panseri et al. used IC-MS to detect and quantify perchlorate, chlorate, and a range of herbicides in baby food commodities.^56^ They demonstrated LLOQ in the range 2–5 ng/mL and precision (%CV) in the range of 5–12%. The reported values for the polar herbicides are 4-fold less than those reported for analysis of a range of polar herbicides in baby food using HPLC-FD (Table 1).^63^

### Environmental Science

The continuous impact of human activities on the environment has resulted in an ever-increasing need for robust and sensitive analytical approaches applied to monitoring the presence of biomarkers and toxic compounds in complex environmental samples. Advances in our understanding of the way the environment, and by extension cellular life, can be negatively impacted, particularly by industrial activities, continuously drive the expansion and updating of regulatory legislation. This is dictated by a greater need for monitoring industrial wastes, e.g., wastewater from pulp or paper mills, or residual pesticides and herbicide levels in food chains. IC-MS has been widely used in environmental sciences since the 1980s in various forms, and its applications have recently been reviewed elsewhere.^64,65^ Here, we focus on a brief history and some of the more recent studies using online electrolytic ion-suppression. Since the mid-1980s, IC was established as the only method for analysis of inorganic anions in environmental samples, and therefore, the development of IC-MS brought new analytical capabilities to already established protocols. Applications included analysis of oxyhalides,^64,66−68^ Cr (VI),^69^ nitrogen- or sulfur-based ions,^70^ and metal–EDTA complexes^71^ (Table 2). While analysis of inorganic chemicals using IC-MS has long been established, analysis of organic molecules in environmental samples has largely been carried out using GC-MS, IP-MS, or HILIC-MS methods.^72^ As discussed earlier, these approaches have their limitations, and the application of IC-MS occurred relatively early in its development from the mid-1990s onward.^73,74^ In 2007, Meyer et al. used ion-suppression technology to develop an IC-MS method for the analysis of aliphatic polyhydroxy carboxylic acids in drinking water and soil leachate.^75^ They characterized 18 different carboxylic acids without postcolumn solvent addition and reported LLODs and LLOQs in the range of 18–60 ng/mL and 45–176 ng/mL, respectively. With postcolumn addition of MeOH, they reported LLODs and LLOQs in the range of 5–119 ng/mL and 12–296 ng/mL, respectively. These values were, in general, lower than those reported using conductivity detection.^75^ In another study, Slingsby et al. developed a method for identification of nine different haloacetic acids in effluent waters with LLOD in the range of 0.1–0.7 ng/mL.^76^ Subsequently, Niu et al. developed an IC-MS method for identification of dialkyl phosphonate acids (DPAs) and hydrolysates of aluminum dialkyl phosphonates (ADPs).^77^ DPAs are formed from hydrolyzation of phosphorus-based flame-retardant ADPs. These methods were used for analysis from tap water, river water, effluent, and influent samples with LLODs and LLOQs in the range of 0.001–0.003 ng/mL and 0.003–0.01 ng/mL, respectively (Table 1). IC-MS was also used for the identification and characterization of molecules with ionic phosphate groups as described by Sjöberg et al.^78^ They estimated a detection limit in the range of 37–99 ng/g. Finally, Zhao et al. recently described the determination of monosaccharides derived from polysaccharides in activated sludge using IC-MS to help understand the mechanism of water treatment.^79^ They showed a LLOD of 0.34–2.15 ng/mL, and %RSDs were 3.76% and 0.27% for peak areas and retention times, respectively.^79^ Using an IC-MS method, they overcame widely reported analytical challenges associated with HILIC-MS, such as column stability and poor retention time reproducibility in the analysis of sugars.^80^ Application of IC-MS in the analysis of environmental samples has clearly demonstrated that it is highly sensitive and robust for the analysis of a wide range of ionic and polar molecules in complex environmental matrices.

### Pharmaceutical Sciences

While IC-MS in forensic science, food chemistry, and environmental science and technology applications has developed relatively quickly, its application in the pharmaceutical sciences has been slower. Developments have focused in three main areas: (i) detection of impurities that result from the synthesis of therapeutics, (ii) identification and characterization of degradation products, and (iii) pharmacokinetics studies. Some early research involving IC-MS, led by Ahrer et al., involved the analysis of degradation products from the cholesterol-reducing drug colesevelam hydrochloride. They were able to characterize compounds not identified by GC-MS.^81^ They demonstrated a detection limit of 10 μg/mL for the standard compound, i.e., hydroxyquat. Second, Corry et al. used IC-MS for the analysis of organic acid impurities in 2-butynoic acid synthesis.^82^ They showed that the relevant organic acid impurities, including acetate, propionate, formate, butanoate, crotonate, and pentanoate, could be measured robustly with high sensitivity. The LLOQ% (ppb) was in the range of 1–5 and RSD% in the range of 4–8. Additionally, the detection limit for most organic acids was 1 ppm. This was a significant improvement on the detection limit of 1–30 μg/mL demonstrated for HILIC-MS analysis (Table 1). Lewis et al. expanded applications to low molecular-weight cationic amines,^83^ which are used as reactants in the chemical synthesis of therapeutics, their analysis being essential for quality control purposes.^83^ They demonstrated analysis of 12 different amines by IC-MS with detection limits (mass of compound on column, measurement of chromatographic peak area) in the range of 0.9–2 ng. The simplified workflow eliminated the need for sample derivatization required by alternative GC-MS and IP-MS methods. Finally, Garcia et al. recently demonstrated the application of IC-MS in pharmacokinetic studies to determine the kinetics of drug elimination in plasma samples.^84^ They analyzed blood plasma from horses for prohibited bisphosphonate drugs, eliminating the need for time-consuming chemical derivatization procedures required by previously applied LC-MS/MS methods. They reported a LLOD of 0.2 ng/mL for zoledronic acid, which was 5-fold less than the previously reported value of 1 ng/mL obtained by other liquid chromatographic methods coupled with mass spectrometry.^85^ More recently IC-MS was used for monitoring potential drug effects in a COVID-19 clinical trial.^86^

### Microbiology

Microbial communities are involved in diverse natural processes linked to health and disease^87−89^ as well as processes such as fermentation in the brewing and wine industries,^90^ crop production,^91,92^ synthesis of raw chemicals,^93^ and wastewater treatment.^94,153^ Many microbially derived metabolites are highly polar or ionic, such as organic acids produced by microbial processes in the gut (e.g., short-chain fatty acids).^95^ The analytical capabilities of IC-MS are therefore theoretically well-suited to applications for monitoring or detecting microbial processes. Surprisingly, to date, only a small number of applications have been published. Notable work reported by Tittle et al. demonstrated application of IC-MS for the analysis of photodegradation products of ^14^C-p-coumaric acid (PCA) as a model of terrestrial dissolved organic carbon (DOC),^96^ because photolysis products of PCA are shown to be similar to those observed from photolysis of natural organic carbon.^96^ Detection limits around 3000 ng/mL were determined for low molecular weight organic acids formed from photodegradation products of PCA. More recently, Glombitza et al. used IC-MS to monitor the impact of fermenting bacterial communities on degradation of high molecular weight organic matter in subseafloor sediments. They measured volatile fatty acids (VFAs) including formate, acetate, and propionate,^97^ which are consumed as electron donors in terminal steps of organic matter mineralization; e.g. sulfate reduction.^97^ While they did not report formal LLODs and LLOQs, they recorded values as low as 0.7 nmol/mL. Other examples of IC-MS in microbial applications include metabolomic analysis of the root endophytic fungus *Piriformospora indica*(98) and analysis of metabolites rhizobia formed with the symbiont *Sesbania herbacea*,^99^ a native North American fast-growing legume. IC-MS has also been combined in a multiomic approach to investigate plasmid maintenance in bacterial communities.^100^Table 3 provides information on selected studies involving IC-MS focused on microbial metabolites with environmental or health impacts.

**Table 3:** List of Secreted Microbial Metabolites with Environmental or Health Impacts^a^

Metabolite (example)	Source	Impact	Ref
Short-chain fatty acids (acetate, propionate, butyrate)	Colonic microbiota fermentation of polysaccharides	Human health; a range of interaction with immune system, pathogenesis of inflammatory bowel disease and cancer	(141−147)
Indole derivatives	Metabolism of tryptophan by intestinal bacteria	Human health, Activation of AhR and NR1I2	(98, 148, 149)
Polyamines (putrescine, spermidine, and spermine)	Metabolism of arginine	Human health, not clear	(98)
Secondary bile acids	Modification of host-produced bile acids by gut microbiota	Human health, activation of GPBAR1 and BAR	(98)
Vitamins like B2, B3, B9	Commensal bacteria	Human health	(150, 152)
Organic acids (acetate and formate)	Surface water microbiota	Mineralization of organic matter in marine sediments	(91)
Lipids	DGDG (34:2), CL (66:3)	Wastewater	(151, 153)
Amino acids	Phenylalanine, Lysine, Tyrosine	Wastewater	(151)

a While most of the molecules are highly polar or have a polar and ionic head group, most of these metabolites have not been studied using IC-MS.

**Figure 4:**
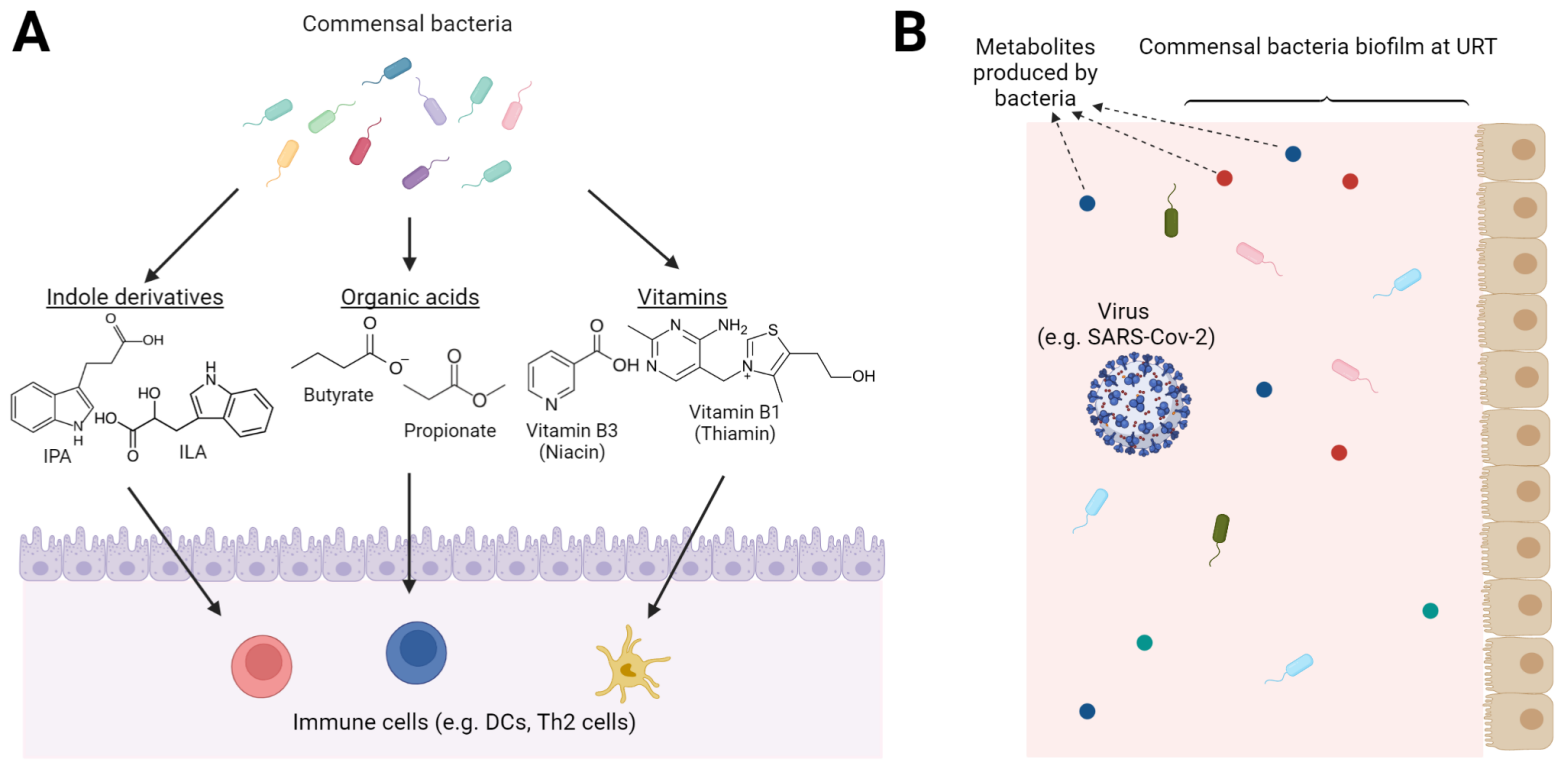
Many metabolites of commensal bacteria affecting human health are highly polar or ionic at physiological pH. (A) Gut bacteria produce different anionic metabolites such as indole derivatives, organic acids, and some vitamins. These metabolites impact different immune cells including dendritic cells (DCs) and Th2 cells. (B) Nasal microbiota in the upper respiratory tract (URT) can potentially interact with the processes of viral infection. Metabolites produced by nasal bacteria may interact with a virus to induce or abolish its infectivity.

The unique analytical capabilities demonstrated by IC-MS provide potential for new investigations into the impact of microbial communities on environmental conditions, e.g. on global cycle of carbon, nitrogen, sulfur, and phosphate, crop production, and industrial processes. An area of particular importance, currently limited from a methodological perspective, is understanding the relationship between microbial metabolism and human health. For example, the influence of gut and nasal bacteria on the human host at the molecular level (Figure 4A and 4B). Highly polar and ionic metabolites, e.g. organic acids, indole derivatizes generated from bacterial metabolism of tryptophan, polyamines, and some vitamins like B3 and B9 play an important role in human health.^101^ There is also a potential role for nasal microbiota in relation to respiratory viral infections.^89,102,103^ Due to the high polarity and ionic nature of many metabolites produced by commensal microbiota, and established relationships with respiratory diseases (e.g., inflammation bowel disease and obesity) we predict that IC-MS has the capability to be an important tool in future studies for discovery and characterization of microbial metabolites that impact human health and immune function.

### Plant Sciences

Plant secondary metabolites are a vast natural resource for the discovery of new natural products with medicinal properties. Many of these molecules are highly polar or negatively charged, and both targeted and untargeted IC-MS applications are receiving increasing interest. In an early study Sekiguchi et al. demonstrated targeted IC-MS analysis of phosphorylated metabolites extracted from seeds of *Arabidopsis thaliana*.^15^ They detected 17 compounds describing robust analysis with detection limits in the range of 10–250 nM and RSD% 93–110%. In another study, Sanchez et al. used an IC-MS/MS method for untargeted analysis of *Ocimum basilicum* (basil) leaves’ metabolome, identifying a range of polar metabolites linked to primary metabolism, e.g., organic acids, monosaccharides, sugar–phosphates, and nucleotides including ATP.^104^ Very recently, Paz et al. demonstrated the application of IC-MS for measuring the level of organic acids in plant root exudates, demonstrating a LLOQ of 5 ng/mL.^105^ The reported LLOQ was significantly lower than those typically reported for HILIC-MS methods (Table 1). Thus, plant science applications, particularly for natural product analysis, highlight the analytical strengths of IC-MS, and we expect applications to continue developing, particularly in relation to improving crop production and discovery of new plant natural products.

### Cell Biology and Metabolomics

The capabilities of HRMS technologies for predicting the molecular formula of small molecules, in combination with advances in bioinformatics and statistical analysis, has enabled increasingly effective characterization of cell extracts and metabolomes.^106,107^ Commonly, HILIC, IPLC, CE, and GC coupled with HRMS have been the chromatographic approaches applied to characterize ionic and polar metabolites, but coverage of some ionic metabolites, and in particular untargeted coverage of highly polar and ionic submetabolomes, remains a major challenge. IC-MS applications using ion-suppression technology in cell biology and metabolomics studies have been increasing in recent years, since the pioneering work of Wang et al.,^108^ which demonstrated IC-MS could be used for the comprehensive analysis of anionic metabolites in head and neck cancer cell extracts.^108^ For example, they demonstrated an approximately 100-fold increase in sensitivity compared to HILIC-MS for a number of metabolites. The reported LLODs for a panel of standard anionic metabolites were 0.04–0.5 pmol/mL with a signal-to-noise ratio of 3. They demonstrated that IC-MS coverage overlapped with UHPLC-MS and HILIC-MS but was able to identify additional metabolites (25 metabolites demonstrated).^108^ This work led to a number of subsequent studies focused on optimization and application of IC-MS for the targeted analysis of highly polar and ionic metabolites.^109−112^ These studies generally reported lower detection limits when compared to HILIC-MS, in the nmol/mL range,^109^ with precision and accuracy in the range of 1–19% and 82–115% respectively.^110^ Studies using IC-MS for the analysis of cell, tissue, and biofluid extracts from a range of organism types have accumulated in recent years.^100,113−123^ These studies represent a mixture of targeted, semitargeted, and untargeted IC-MS applications and collectively demonstrate the efficacy and benefits that IC-MS can bring to the analysis of cell extracts and metabolomics studies. Studies from the author’s lab have demonstrated the application of IC-MS for untargeted analysis, particularly for the coverage of metabolites linked to central carbon metabolism in cells. For example, we applied IC-MS/MS to measure low levels of the naturally occurring nucleotide analogue ddhCTP (3′-deoxy-3′, 4′-didehydrocitidine triphosphate) formed by the antiviral enzyme SAND (previously RSAD2 (viperin)).^124−154^ Our untargeted metabolomic analysis using samples extracted from human induced pluripotent stem cells (iPSCs)-derived macrophages revealed a function of ddhCTP in immunometabolism.^126^ In a separate study we developed a modified IC-MS/MS method for untargeted metabolomics and characterized over 400 endogenous human metabolites, demonstrating the stability and reproducibility of the method and its benefits in terms of compound coverage compared directly to HILIC-MS.^127^ Investigating altered metabolism linked to isocitrate dehydrogenase one (IDH1) mutations in cancer showed links with specific changes in lysine and tryptophan metabolism as well as altered β-citryl-glutamate, N-acetylated amino acids, and other amino acid derivatives.^127^ As the availability of IC-MS systems increases, its complementary capabilities for robust targeted and untargeted analysis of complex biochemical extracts will likely lead to increasing applications in a wider range of metabolomics and cell-based studies.

## Perspective and Concluding Remarks

**Figure 5:**
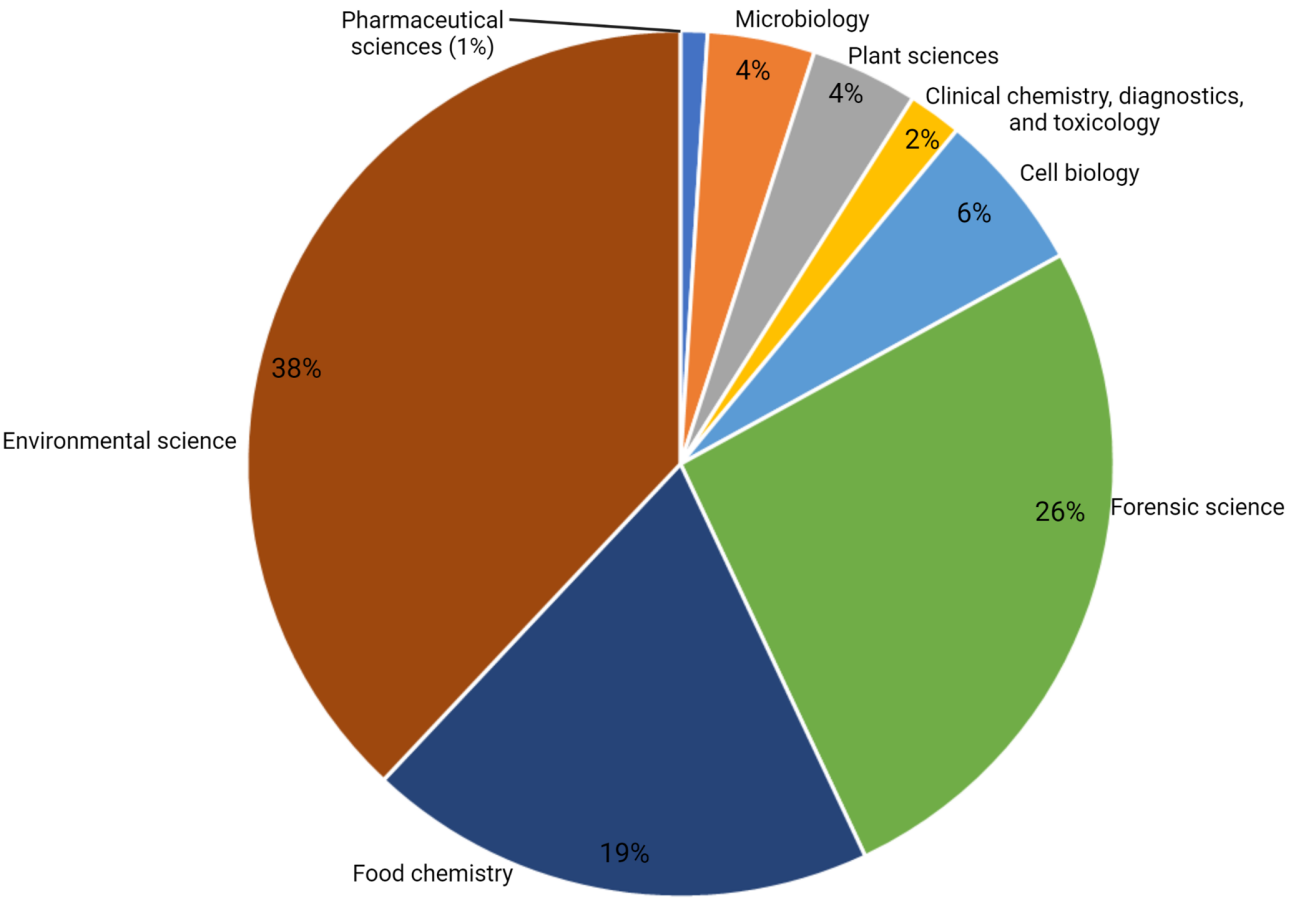
Graph illustrating the different research areas where IC-MS has been applied to date with an estimate of the proportion of papers published up to 2021 (based on a Google Scholar search). Recent studies have shown that IC-MS has significant potential in biological and medicinal research applications, an area of IC-MS application expected to grow in the future.

The development of ion-suppression technology, particularly continuous electrolytic ion suppression, has enabled the hyphenation of ion-exchange chromatography with high-resolution mass spectrometry, a combination that has brought new analytical opportunities. Commercialization of IC-MS platform technology has seen an increasing number of laboratories exploring new application areas for IC-MS and has revealed successes beyond traditional application areas. For example, in addition to analysis of inorganic ions, organic and biological polar and ionic analytes have been successfully targeted and characterized in a wide range of environmental and biological sample types. A combination of eluent generation and polarity selectivity, inherent to IC-MS analysis using ion-suppression, decreases effective matrix complexity, reducing the potential for matrix effects and chromatographic crowding that can lead to analytical interference using mass spectrometry detection. Analytes are often already in ionic form; therefore, high sensitivity in analysis by mass spectrometry detection can be achieved with minimal ion suppression. In contrast, alternative chromatographic approaches for ionic and highly polar analyte characterization (e.g., RP-MS, HILIC-MS, GC-MS, and IP-MS) can suppress the ionic characteristics of analytes (use of low protic solvents, derivatization, etc.) to facilitate effective analysis conditions which can lead to a bias in coverage and signal suppression. In summary, IC-MS has emerged as an effective complementary (or alternative) analytical tool, demonstrating high levels of platform stability, retention time reproducibility, sensitivity, and low limits of detection. Most applications to date have focused on forensic science, environmental science, technology and manufacturing, and food chemistry. However, applications in pharmaceutical sciences, clinical chemistry settings, diagnostics, microbiology, metabolomics, and cell biology are increasingly being seen, and there is room for significant further developments and applications in these areas (Figure 5).

There is scope for new IC-MS applications wherever analytes are highly polar or ionic, including those embedded in complex matrices. To indulge in speculation, we suggest future IC-MS applications will include a significant increase in the investigation of complex biological and environmental systems and processes, host–pathogen relationships, microbiome metabolism, relationships between plant and soil chemistry, pharmacokinetics and dynamics, and biomarker studies related to diagnosis, prognosis, and etiology of disease. Traditionally these areas are particularly challenging analytically, especially using untargeted approaches; IC-MS therefore has the potential to make important contributions in both discovery-orientated and targeted applications.
